# Managing product variety on online platform: Consumer heterogeneity and diseconomies of scope

**DOI:** 10.1371/journal.pone.0219177

**Published:** 2019-08-12

**Authors:** Ganfu Wang, Xingzheng Ai, Li Zhong

**Affiliations:** 1 School of Business Administration, Hubei University of Economics, Wuhan, China; 2 School of Management and Economics, University of Electronic Science and Technology of China, Chengdu, China; Harbin Institute of Technology, CHINA

## Abstract

Recent innovations in e-commerce have led to the emergence of online retailing platforms, where millions of products are sold. Most of these products are sold by third-party sellers who pay a fee for the e-retailer (called the platform owner). We investigate how the e-retailer manages the various products in the presence of consumer heterogeneity and diseconomies of scope. Our analytical results indicate that the e-retailer prefers the platform-selling mode when consumers have stronger heterogeneity or when the value of a product is high; moreover, the consumer heterogeneity benefits the e-retailer and hurts the supplier. We also analyze the effect of the relationship of among categories on the e-retailer’s choice. We show that the relationship among categories can invert the existing format. In addition, we find that the e-retailer may be better off and raise the number of products under strong diseconomies of scope when the categories are complements, and the opposite is true when the categories are substitutable.

## 1. Introduction

The rapid development of information technologies has provided a new channel for consumers to shop. It has resulted in online retail sales totaling Ұ7.17 trillion in 2017 and accounting for 15% of total retail sales. E-retailers who have traditionally acted as resellers are starting to offer online platform services providing a direct connection between buyers and sellers. In this selling mode, the e-retailers allow hundreds of thousands of manufacturers (also known as independent sellers) direct access to their customers while charging a fee for providing this access. This format is called the platform mode. On online platforms, consumers can observe the volume of sold products. When a product has higher sales, more consumers buy it, and the product’s value will be growing with the numbers of users. This is called the direct network effects. When the number of users becomes larger on one side, the number of suppliers also becomes larger on the other side. To elaborate, the platform has two-sided effects in the sense that both sides-consumers and suppliers need to obtain access to the same platform in order to interact. Furthermore, when the value of the platform to one side is higher, more members will be present on the other side. This is known as the indirect network effects. (e.g., Armstrong.[1], Rochet and Tirole.[2]). That is, consumers’ values from joining a platform increase with levels of product variety. Meanwhile, manufacturers’ profits from joining the platform increase with the number of consumers that have adopted it. (Wely.[3]). The more categories that there is, the more attractive the platform is. In other words, consumers are heterogeneous for their willingness to pay for products and services (Shi et al.[4]). However, the managing costs of product variety increase with the number of product categories (David.[5]), due to the effects of diseconomies of scope. The higher variety level often incurs higher marginal variety costs, which leads to the high costs to expand the variety. Therefore, how many categories are best for the platform owner?

For example, direct sales from suppliers accounted for 45% of units sold on Amazon.com in the second quarter of 2015. Similarly, JD.com was founded in 1998 as a pure reseller, and then it aggressively expanded its platform offerings. By 2014, JD was a hybrid mode, with sales roughly evenly distributed between the reselling mode and platform mode. In 2014, JD itself sold only 6.28% of the products listed on its online platform. Therefore, which products are sold in reselling modes and which ones are left to the third–party sellers?

Given the above, how to manage multi-categories is a critical issue for the platform owner. On the one hand, product variety meets consumers’ preferences and improves market liquidity. On the other hand, the management costs of multi-categories increase with the variety due to the diseconomies of scope. Platform owners need to balance product variety and management costs of categories to maximize their profits. Therefore, we investigate how to govern multi-categories on online platforms by addressing the following questions. For a platform owner, which categories are sold by platform modes and which ones are sold by reselling modes in the presence of consumer heterogeneity and diseconomies of scope? What are the key drivers of that determination? What are the optimal number of categories for the platform owner? How do the relationships among categories affect the platform owner’s performance? We establish a formal model to analyze some of the basic problems regarding which categories to offer under each mode.

## 2. Literature review

The economics literature on two-sided markets focus on pricing strategies (e.g., Armstrong [1], Rochet and Tirole [2] 2003, Parker and Alstyne [6]). This literature primarily focuses on cross network effects and has addressed the impact of the intensity of indirect network effects, demand elasticities, and coordination issues on the pricing structures of platforms. Following [2], Wey [3] develops a general theory of a monopoly pricing of multi-sided platforms, in which platforms use insulating tariffs to avoid coordination failures and implement any desired allocation. Kung and Zhong [7] focus on pricing strategies, including membership-based pricing, transaction-based pricing, and cross-subsidization. Hagiu [8] explains that the consumers’ preferences for product variety are a key factor in determining the optimal pricing structures of platforms where the level of variety is exogenous. However, in our paper, we study operating modes of multi-categories of platforms under the diseconomies of scope and consumer heterogeneity where the level of variety is endogenous. Besides, Reisinger [9] studies two-part tariff (a subscription and a per-transaction fee) in competition two-sided platforms. Ryan et al. [10] explore whether the retailer should sell products on an online platform which sells products., and they model a contract between the platform owner and the retailer with a fixed fee. Mantin et al.[11] investigate when does the retailer introduce a third-party marketplace. They show that the retailer who runs a third-party marketplace creates an “outside option”, which improves bargaining power in negotiations. Unlike these papers, we identify the condition in which the e-retailer chooses the reselling mode or the platform mode in the presence of consumer heterogeneity and diseconomies of scope when the number of categories is endogenous.

Our work is related to the literature on product variety management and strategic choice in a multi-sided platform. There is an extensive literature on managing product variety (e.g., Cachon and Kok[12], Smith and Agrawal[13], Rajagopalan and Xia [14]). These papers focus on the optimal inventory level. However, our focus is on the operating modes of categories. In addition, we take the direct and indirect network effects into account. Jiang et al.[15] use game theory to analyze the strategic interaction between the platform owner and the independent seller. The platform owner can track the seller’s sales to identify the product’s demand volume and then sells high demand products by itself. The results show that regardless of the low-demand and the high-demand of products, sellers may benefit from the platform owner’s entrance. However, they do not incorporate cross-group effects and direct network effects, which are essential features of the platform, and they also do not consider the effects of diseconomies of scope. Hagiu and Wright [16] analyze an intermediary’s choice between as a marketplace, as a reseller, or as a hybrid. The model consists of an intermediary and N independent suppliers and the private information is different between the suppler and intermediary. Moreover, they emphasize that the fundamental distinction between marketplaces and resellers is the control rights. Meanwhile, our model is different from them in three aspects. First, here we consider the impact of the diseconomies of scope and consumer heterogeneity on the platform owner’s strategies, while they does not consider this. Second, we focus on category management, and the number of categories is taken as a decision variable that affects the platform owner’s strategies but that is exogenous in Hagiu and Wright [16]. Third, we also analyze the impact of substitutes and complements of products on the platform owner’s performance.

We have found a few works in multi-sided platforms governance. Hagiu.[17] qualitatively analyze governance rules for a multisided platform and shows two key categories: the first is the rules regulating the access to the multisided platform, and the second is rules regulating the interactions on the multisided platform. Casadesus-Masanell and Halaburda [18] construct a model of two-sided platforms by connecting users with complementary products such that platforms may have an incentive to limit the number of complementary products. Hagiu [19] explores the factors that drive platforms to restrict access. He shows that users’ values depend on both the number of other users who join the platform and their average quality. Wang and Ai [20] investigate how to manage product variety on the platform in the presence of product market risk and scope economies. Unlike these works, we focus on the operating modes of multi-categories under diseconomies of scope and consumer heterogeneity. Furthermore, the number of categories is a decision variable in our model. In addition, we analyze the impact of the relationship of among categories on the platform owner’s operating strategies.

The contributions of this work are as follows: First, our article identifies the impact of consumer heterogeneity and diseconomies of scope on the platform owner’s operating strategy. To be more specific, we establish conditions under which the categories are adopted the reselling mode, and under which categories are adopted the platform mode. Second, we develop an optimal number of product categories that maximizes the platform’s profits r. Third, we reveal the impact of the relationships of among categories on the platform performance and the optimal number of product categories. Fourth, we identify the impact of consumer heterogeneity and diseconomies of scope on the platform access fee.

The remainder of the paper is organized as follows. In the next section, we describe the model. In section 4, we report the optimal choice of platform owners. In section 5, we discuss the extent to which the main results extend to the case of substitutable or (complementary) relationships between products. In section 6, we present the numerical analysis. In section 7, we conclude with some shortcomings of our research along with directions for future work.

## 3. Model Setting

By considering the product variety available on a monopoly e-retailer with an online platform (also called platform owner), the e-retailer not only sells products directly but also allows hundreds of thousands of independent manufacturers to sell products on its online platform. There are *n* independent manufacturers. The manufacturer *i*(*i* = 1,2,⋯*n*) supplies a unique product category and the quantity is *q*_*i*_. The product’s value is heterogeneous for different consumers. *ε*_*i*_ is an independent random variable with a normal distribution *N*(0,*σ*_*i*_^2^) that represents the gap between an individual’s actual value, where *σ*_*i*_^2^ denotes the degree of consumer heterogeneity. In our model, all product categories are independent and heterogeneous, and manufacturers are symmetrical. Then, we set *ε*_*i*_ = *ε*, *σ*_*i*_^2^ = *σ*^2^ and *q*_*i*_ = *q*. Following Hagiu[21], we incorporate direct network effects and use a linear model. Our model implicitly assumes that sellers set a single market clearing price. Therefore, the inverse demand function is

$$p_{i}=a+\varepsilon +\beta q-q$$

Where *a* represents product value per-unit, and *β*∈(0,1) is the degree of direct network effects. As the product variety increases, the e-retailer grows its management costs (Xia and Rajagopalan[22], 2009). To focus on the effects of diseconomies of scope, we normalize the marginal production costs to zero. Similar to Xiao et al [23]. (2015), we assume that the total management costs of products with the variety of *n* for the e-retailer is

$$\begin{array}{l} C(n,q_{1},q_{2}\ldots q_{n})=nf+\frac{1}{2}\sum_{i=1}^{n}\sum_{j=1}^{n}rq_{i}q_{j}\\ \;=nf+\frac{1}{2}\gamma (nq{)}^{2} \end{array}$$

where *f* is the marginal variety cost for increasing the variety, which is identical for the e-retailer and the manufacturer. *γ* is the externality cost factor and *γ*>0 represents the degree of diseconomies of scope. Diseconomies of scope happen due to the negative externalities in the management process, such as increasing inventory costs. Subscripts *r* and *m* denote the e-retailer and the manufacturer, respectively.

## 4. Modes analysis

In this section, we analyze which selling formats will be adopted by the e-retailer in the presence of diseconomies of scope and consumer heterogeneity. To characterize the relationship between consumer heterogeneity and profits, we use expected profits to express each player’s profits.

### 4.1. Reselling mode

In this format, first, the e-retailer directly sells products (i.e., reselling) and sets the optimal number of product categories. Second, the manufacturer *i* sets its wholesale price *w*_*i*_. After manufacturers set its terms, the e-retailer procures *q* units for each category and sells to end consumers. The effects of diseconomies of scope incur when the e-retailer manages multi-categories. Therefore, the e-retailer’s profit function can be written as

$$\pi_{r}=n(a+\varepsilon +\beta q-q-w_{i})q-nf-\gamma {\left(nq\right)}^{2}/2$$ (1)

The online platform has advanced information technologies, and the e-retailer who can obtain consumers’ information makes decisions based on consumers’ states. However, manufacturers do not have that and use the expected quantity to make decisions. Then, the manufacturer’s profit function is

$$\pi_{mi}=w_{i}Eq$$ (2)

We solve this subgame using backward induction. The market equilibrium quantity and the wholesale price under this situation are as follows

$$q=\frac{a+\varepsilon }{2(2-2\beta +n\gamma )},\;w_{i}=\frac{a}{2}$$ (3)

The e-retailer’s expected profits are

$$E\pi_{r}=\frac{n(a^{2}+4\sigma^{2}+8f(2-2\beta +n\gamma ))}{8(2-2\beta +n\gamma )}$$ (4)

The e-retailer selects the number of categories that maximize its expected profits in the presence of diseconomies of scope and consumer heterogeneity. Therefore, the optimal number of product categories is

$$n^{*}=\frac{\sqrt{\left(1-\beta )f(a^{2}+4\sigma^{2}\right)}-4(1-\beta )f}{2f\gamma }$$ (5)

Then, the e-retailer’s optimal expected profits are

$$E\pi_{r}=\frac{{\left(\sqrt{a^{2}+4\sigma^{2}}-4\sqrt{(1-\beta )f}\right)}^{2}}{8\gamma }$$ (6)

The manufacturer *i*‘s expected profits are

$$E\pi_{mi}=\frac{a^{2}f}{2\sqrt{(1-\beta )(a^{2}+4\sigma^{2})f}}$$ (7)

### 4.2. Platform modes

In this situation, manufacturers directly sell to consumers on the online platform and obtain the consumers’ information. Therefore, manufacturers make decisions based on consumers’ states.

#### 4.2.1. Fixed fee

In this format, each manufacturer maintains control rights over their marketing decisions *q* and pays a fixed access fee *F*_*r*_>0 to the e-retailer (the platform owner) for joining its the online platform. First, the e-retailer charges the fixed access fee. Then, the manufacturers can directly sell to consumers on online platform after accepting the access fee. Moreover, each manufacturer incurs a fixed management cost *f* for products. Superscript *PF* denotes the fixed access fee in the platform mode. In this situation, the profit function of the manufacturer *i* can be written as

$$\pi_{mi}^{PF}=(a+\varepsilon +\beta q-q)q-f-F_{r}$$ (8)

The e-retailer’s profit function is

$$\pi_{r}^{PF}=nF_{r}$$ (9)

We observe the following quantity in this selling format:

$$q_{}^{PF}=\frac{a+\varepsilon }{2(1-\beta )}$$ (10)

By substituting Eq (10) into Eq (8), manufacturer *i*‘s profits can be written as follows:

$$\pi_{mi}^{PF}=\frac{{\left(a+\varepsilon \right)}^{2}-4(1-\beta )f}{4(1-\beta )}-F_{r}$$ (11)

In the platform mode, the e-retailer must ensure that each manufacturer’s expected profits are no less than that in the reselling mode. Otherwise, the manufacturers will still choose the reselling mode. Therefore, there is a sufficient condition to ensure this property

$$E\pi_{mi}^{PF}\geq E\pi_{mi}^{}$$ (12)

According to (12), we obtain the fixed access fee as follows:

$$F_{r}=\frac{a^{2}+\sigma^{2}-4(1-\beta )f}{4-4\beta }-\frac{a^{2}\sqrt{(1-\beta )f}}{2(1-\beta )\sqrt{a^{2}+4\sigma^{2}}}$$ (13)

The e-retailer sets the uniform fixed fee for all manufacturers regardless of consumer heterogeneity. If the fixed fee is sufficiently high, not all manufacturers will join the online platform. All manufacturers choose the platform mode if and only if the e-retailer charges the fixed access fee without any differences. Therefore, the e-retailer’s expected profits from participating with the platform mode is

$$E\pi_{r}^{PF}=\frac{\left(\sqrt{a^{2}+4\sigma^{2}}-4\sqrt{(1-\beta )f})[{\left(a-\sqrt{(1-\beta )f}\right)}^{2}-5(1-\beta )f\right]}{8\gamma \sqrt{(1-\beta )f}}$$ (14)

#### 4.2.2. Profits sharing

In reality, the e-retailer may choose profit sharing contracts as the platform access fee. Assuming that the shared proportion for the e-retailer is *t*, correspondingly, the manufacturer’s proportion is 1−*t*. Superscript *PT* denotes the situation of profit sharing in the platform mode. Then, the profits of the e-retailer and manufacturer *i* can be written as follows:

$$\begin{array}{l} \pi_{r}^{PT}=\frac{nt[{\left(a+\varepsilon \right)}^{2}-4(1-\beta )f]}{4(1-\beta )}\\ \pi_{mi}^{PT}=\frac{\left(1-t)[{\left(a+\varepsilon \right)}^{2}-4(1-\beta )f\right]}{4(1-\beta )} \end{array}$$ (15)

Similarly, to guarantee that the profits of all manufacturers are no less than that in the reselling mode, we have the sufficient condition as follows:

$$E(\pi_{mi}^{PT})\geq E\pi_{mi}^{}$$ (16)

Then, we get the threshold proportion of profit sharing:

$$t=1-\frac{2a^{2}\sqrt{(1-\beta )f}}{\sqrt{a^{2}+4\sigma^{2}}[a^{2}+\sigma^{2}-4f(1-\beta )]}$$ (17)

By substituting (17) into the e-retailer’s profit function, the e-retailer’s expected profits are:

$$E\pi_{r}^{PT}=\frac{\left(\sqrt{a^{2}+4\sigma^{2}}-4\sqrt{A})[(a^{2}+\sigma^{2}-4A)\sqrt{a^{2}+4\sigma^{2}}-2a^{2}\sqrt{A}\right]}{8\gamma \sqrt{A(a^{2}+4\sigma^{2})}}$$ (18)

Notes *A* = (1−*β*)*f*.

Now we analyze the related partial derivatives of *t* as follows:

$$\frac{\partial t}{\partial \sigma^{2}}=\frac{2a^{2}\sqrt{(1-\beta )f}(3a^{2}+6\sigma^{2}-8(1-\beta )f)}{{\left(a^{2}+\sigma^{2}-4(1-\beta )f\right)}^{2}{\left(a^{2}+4\sigma^{2}\right)}^{3/2}}>0.$$ (19)

$$\frac{\partial t}{\partial \beta }=\frac{a^{2}\sqrt{f}(a^{2}+\sigma^{2}-4(1-\beta )f)}{\sqrt{\left(1-\beta )(a^{2}+4\sigma^{2}\right)}{\left[a^{2}+\sigma^{2}-4(1-\beta )f\right]}^{2}}>0.$$ (20)

We summarize the impact of the degree of consumer heterogeneity (*σ*^2^) and the direct network effects (*β*) on the sharing proportion (*t*) in the proposition below.

**Proposition 1** The sharing proportion of the e-retailer (*t*) increases with consumer heterogeneity (*σ*^2^) and direct network effects (*β*) in the platform mode.

Proposition 1’s implication: When the degree of consumer heterogeneity is high, consumer segments are full. When the direct network effects are strong, more consumers purchase products. Thus, the manufacturer acquires higher expected profits. Therefore, in order to acquire higher expected profits, manufacturers must share profits with the e-retailer. Because the heterogeneous consumers incur price fluctuations and operating risks, manufacturers decline the operating risks by sharing profits with the e-retailer. It is a benefit for system to gain higher profits and each participant also obtains higher expected profits. However, the e-retailer extracts manufacturers’ profits by improving the proportion of profit sharing. Therefore, the proportion of profit sharing is high.

Now, we investigate the impact of diseconomies of scope and consumer heterogeneity on the e-retailer’s expected profits. The following proposition answers this problem.

**Proposition 2** The e-retailer’s expected profits increase with consumer heterogeneity (*σ*^2^), but decrease with diseconomies of scope (*γ*).

Since different consumers are willing to pay different prices for the products, then manufacturers extract more consumer surplus and gain higher expected profits. Essentially, the e-retailer who owns the online platform extracts manufacturers’ additional surplus from the platform mode beyond the reselling mode. Therefore, as the degree of heterogeneity (*σ*^2^) increases, the e-retailer earns higher expected profits. However, the management costs of products and transaction costs increase with the degree of diseconomies of scope. Therefore, the e-retailer’s expected profits decrease when the degree of diseconomies of scope is increasing.

Facing the fixed fee and profits sharing, which one is better for the e-retailer? The following proposition answers this problem.

**Proposition 3** The e-retailer prefers profits sharing for the platform access fee when consumers are heterogeneous in the platform mode.

According to proposition 1, the manufacturers have higher the operating risk when the degree of consumer heterogeneity is higher. Subsequently, manufacturers share operation risks and profits with the e-retailer who obtains higher expected profits by profit sharing. However, the e-retailer will not share the operating risks if the e-retailer chooses a fixed access fee. Then, manufacturers have not motivated to improve operating efficiency when consumers are heterogeneous, and the e-retailer gets lower expected profits. Therefore, the e-retailer designs profit sharing to improve operating efficiency when there is consumer heterogeneity.

### 4.3. Choosing modes analysis

In this subsection, we examine how the e-retailer chooses operating modes. For the e-retailer, which categories are adopted the platform mode and which categories are adopted the reselling mode. This choice is specified in the following proposition. See the Appendix proof.

**Proposition 4** The e-retailer prefers the platform mode when $0<a<4\sqrt{(1-\beta )f}$ and *σ*^2^>4(1−*β*)*f*−*a*^2^/4, or $a>4\sqrt{(1-\beta )f}$.

Proposition 4’s implication: the category is adopted the platform mode when the category’s value is small and the degree of consumer heterogeneity is high or the category’s value is large. Since the number of product categories is larger when the degree of consumer heterogeneity is higher. Then, due to effects of diseconomies of scope, the management costs of product categories increase if the e-retailer itself sells. Furthermore, the e-retailer that itself directly manages the higher values of product categories has higher operating risks if the e-retailer since the retail price has greater variation (*σ*^2^ is higher). To reduce operating risks, the e-retailer adopts the platform mode in which the double marginalization effects and the e-retailer’ management costs are relieved. This proposition reveals that the e-retailer chooses the platform mode in the presence of a higher degree of consumer heterogeneity or higher values of product categories.

We next address how the diseconomies of scope and direct network effects affect the optimal number of categories. We can summarize the results in the following proposition.

**Proposition 5** The optimal number of categories is increasing with the consumer heterogeneity (*σ*^2^) but decreasing with diseconomies of scope (*γ*). However, it is increasing with direct network effects (*β*) when the consumer heterogeneity is weak, whereas it is decreasing with direct network effects when the degree of consumer heterogeneity is strong.

When the degree of consumer heterogeneity is high, the consumers prefer product variety. The e-retailer establishes a large number of categories to meet the demands of consumers. In addition, the e-retailer extracts consumer surplus by setting different prices for heterogeneous consumers and obtains higher expected profits. However, the management costs of product variety and transaction costs increase with the degree of diseconomies of scope. To increase expected profits, the e-retailer will decrease the number of categories to reduce the costs of managing multi-products. In addition, the sales volume increase with the direct network externalities so that the e-retailer’ expected profit is increasing. Due to the strong direct network effects, the e-retailer reduces the number of categories to decrease the management costs of the product variety. Since the direct network effects enhance consumers’ attractiveness and the e-retailer’s expected profits; Moreover, it is beneficial to reduce the product variety level and the management costs of product variety.

## 5. The impact of the relationship of among categories on e-retailer’s strategies

In this section, we focus on the impact of the relationship among product categories on the e-retailer’s strategies. To keep things as simple as possible, in our analysis, we assume *k*∈(-1,1) in order to accommodate different interpretations. This model denotes that the relationship of product categories is complementary when *k* is negative and substitutable when *k* is positive. Specially, it is independent when *k* is zero. Superscript *s* denotes the relationship among categories. To secure positive profits, the following conditions must stand: 1−*β*>*k*>−*γ*/2, and $\sigma^{2}>4\sqrt{f(1-\beta -k)}-a^{2}/4$. When the e-retailer chooses the reselling mode, the profit function of the e-retailer and each manufacturer are respectively

$$\pi_{r}^{s}=n(a+\varepsilon +\beta q-q-k(n-1)q-w_{i})q-nf-\gamma {\left(nq\right)}^{2}/2$$ (21)

$$E\pi_{mi}^{s}=w_{i}Eq$$ (22)

The equilibrium quantity and wholesale price are respectively

$$q=\frac{a+2\varepsilon }{4-4\beta +4k(n-1)+2nr},\;w_{i}=\frac{a}{2}$$ (23)

Then, the expected profits of the e-retailer are

$$E\pi_{r}^{s}=\frac{n(a^{2}+4(\sigma^{2}+2f(-2+2\beta +2k-2kn-n\gamma )))}{8(2-2\beta -2k+2kn+n\gamma )}$$ (24)

When the e-retailer obtains the maximum expected profits, the optimal number of product categories is

$$n^{**}=\frac{\sqrt{\left(a^{2}+4\sigma^{2})f(1-\beta -k\right)}-4f(1-\beta -k)}{2f(2k+\gamma )}$$ (25)

The expected retail price and quantity are respectively

$$Ep^{s}=\frac{a((2\gamma +3k)\sqrt{\left(a^{2}+4\sigma^{2}\right)}-2\gamma \sqrt{f(1-\beta -k)})}{(4k+2\gamma )\sqrt{\left(a^{2}+4\sigma^{2}\right)}}$$ (26)

$$Eq^{s}=\frac{af}{\sqrt{\left(a^{2}+4\sigma^{2})f(1-\beta -k\right)}}$$ (27)

Then, the expected profits of the e-retailer and each manufacturer are respectively

$$E\pi_{r}^{s}=\frac{{\left(\sqrt{a^{2}+4\sigma^{2}}-4\sqrt{f(1-\beta -k)}\right)}^{2}}{8(2k+\gamma )},\;E\pi_{mi}^{s}=\frac{a^{2}\sqrt{f(1-\beta -k)}}{2(1-\beta -k)\sqrt{a^{2}+4\sigma^{2}}}$$ (28)

We examine the impact of the relationships of among product categories on the e-retailer’s expected profits and manufacturers’ expected profits. The following proposition answers this question.

**Proposition 6** The e-retailer’s expected profits are decreasing in *k* when $0<\gamma <\bar{\gamma }$. However, the manufacturer’s expected profits are increasing in *k* regardless of the degree of diseconomies of scope.

Notes $\bar{\gamma }=\frac{\sqrt{\left(a^{2}+4\sigma^{2})f(1-\beta -k\right)}-4f(1-\beta )}{2f}$.

Proposition 6 shows: in reselling modes, as the relationship of categories from complementary to substitutable, the e-retailer is worse off when the degree of diseconomies of scope is sufficiently low, but the manufacturer is better off. This is because the quantity of each category is decreasing and the retail price is increasing when the products are complementary. Therefore, the number of categories is larger on the online platform. It is beneficial for the e-retailer but hurts manufacturers. However, the opposite is true when the products are substitutes.

We now address when does the e-retailer choose the platform mode and charges the platform access fee. According to section 4, the e-retailer prefers profit sharing for the platform access fee when consumers are heterogeneous. Therefore, in this section, the e-retailer adopts profits sharing for the platform access fee. Let *t*^*s*^ be the profit sharing proportion of the e-retailer and the manufacturer’s profit sharing proportion is 1−*t*^*s*^. In the platform mode, the profits functions of the e-retailer and each manufacturer are respectively given by the following:

$$\pi_{mi}^{Ps}=(1-t^{s})[(a+\varepsilon +\beta q-q-k(n-1)q-w)q-f]$$ (29)

$$\pi_{r}^{Ps}=nt^{s}[(a+\varepsilon +\beta q-q-k(n-1)q-w)q-f]$$ (30)

We acquire the expected quantity and the retail price as follows

$$Eq^{Ps}=\frac{af(2k+\gamma )}{2(1-\beta -k)f\gamma +k\sqrt{\left(a^{2}+4\sigma^{2})f(1-\beta -k\right)}}$$ (31)

$$Ep^{Ps}=\frac{a}{2}$$ (32)

Then, the proportion of profit sharing and the e-retailer’s expected profits are respectively as follows:

$$t^{s}=\frac{\sqrt{a^{2}+4\sigma^{2}}((2k+\gamma )\sigma^{2}+(\gamma +k)a^{2})-2a^{2}\gamma \sqrt{B}}{(2k+\gamma )(a^{2}+\sigma^{2})\sqrt{a^{2}+4\sigma^{2}}},$$ (33)

$$E\pi_{r}^{Ps}=\frac{\begin{array}{l} (\sqrt{a^{2}+4\sigma^{2}}-4\sqrt{B})[\sqrt{a^{2}+4\sigma^{2}}((2k+\gamma )\sigma^{2}+\\ \left(\gamma +k)a^{2}-4\gamma B)-2a^{2}(k+\gamma )\sqrt{B})-8k\sigma^{2}\sqrt{B}\right] \end{array}}{4(2k+\gamma )((a^{2}+4\sigma^{2})k+2\gamma \sqrt{(a^{2}+4\sigma^{2})B})}$$ (34)

Notes *B* = *f*(1−*β*−*k*).

Compared to the reselling mode, the retail price is lower and the quantity is higher when the categories are substitutable in the platform mode. However, the quantity is lower when the categories are complementary in the platform mode. Although the double marginalization effects are moderated in the platform mode, the quantity is affected by the relationships of categories.

We examine the impact of the relationships of product categories on the e-retailer’s strategies and summarize the results in the following proposition.

**Proposition 7** (1) When the categories are substitutable (*k*>0), the e-retailer always prefers the platform mode ($E\pi_{r}^{Ps}>E\pi_{r}^{s}$).

(2) When the categories are complements (*k*<0), the e-retailer prefers the platform mode ($E\pi_{r}^{Ps}>E\pi_{r}^{s}$) if $\gamma >-k\sqrt{\left(a^{2}+4\sigma^{2}\right)}/\sqrt{B}$; otherwise, the e-retailer prefers the reselling mode if $0<\gamma <-k\sqrt{\left(a^{2}+4\sigma^{2}\right)}/\sqrt{B}$.

See the Appendix.

Proposition 7 shows the following. Under condition 1, the e-retailer always prefers the platform mode regardless of the degree of consumer heterogeneity and diseconomies of scope when the categories are substitutable. The reasons are similar to proposition 4. Under condition 2, when the categories are complements, if the degree of diseconomies of scope is strong, the e-retailer gets higher expected profits in the platform mode. However, if the degree of diseconomies of scope is weak, the e-retailer gets higher expected profits in the reselling mode. This is because the management costs of product variety are higher when the degree of diseconomies of scope is strong. The e-retailer who resells products to consumers incurs higher management costs of product variety. Therefore, the e-retailer adopts the platform mode to maximize its expected profits. In contrast, when the degree of diseconomies of scope is weak, the management costs of product variety are lower. It is a benefit for the e-retailer to adopt the reselling mode since the managing costs are lower and the products are complements. That is, whether the platform mode or the reselling mode is preferred, which depends on the relationships of among categories and the degree of diseconomies of scope.

Now, we analyze the impact of the relationship of among categories, the degree of consumer heterogeneity and the degree of diseconomies of scope on the optimal number of categories, and we summarize this outcome in the following proposition.

**Proposition 8** (1) When the categories are substitutable (1−*β*>*k*>0);

- If the product value is small ($4\sqrt{A}<a<4(\sqrt{A}+\sqrt{B})$) and the degree of consumer heterogeneity is high ($\sigma^{2}>{\bar{\sigma }}^{2}$), then n**<n*. When the degree of consumer heterogeneity is low ($0<\sigma^{2}<{\bar{\sigma }}^{2}$) and the degree of diseconomies of scope is weak ($0<\gamma <\frac{2k\sqrt{A}(\sqrt{a^{2}+4\sigma^{2}}-4\sqrt{A})}{4fk-\sqrt{a^{2}+4\sigma^{2}}(A-\sqrt{B})}$), then n**<n*.
- If the product value is high ($a>4(\sqrt{A}+\sqrt{B})$), then n**<n* regardless of the degree of consumer heterogeneity (*σ*^2^) and the degree of diseconomies of scope (*γ*).
  (2) When the categories are complements ($-\frac{\gamma }{2}<k<0$)

- If the product value is small ($4\sqrt{B}<a<4(\sqrt{A}+\sqrt{B})$)
  When the degree of consumer heterogeneity is high ($\sigma^{2}>{\bar{\sigma }}^{2}$), then n**>n*.
  When the degree of consumer heterogeneity is low ($0<\sigma^{2}<{\bar{\sigma }}^{2}$), if the degree of scope diseconomies $\gamma \in (0,\frac{-2k(\sqrt{A(a^{2}+4\sigma^{2})}-4A)}{\sqrt{\left(a^{2}+4\sigma^{2}\right)}(\sqrt{A}-\sqrt{B})-4fk})$, then n**>n*.
- If the product value is large ($a>4(\sqrt{A}+\sqrt{B})$), then n**>n* regardless of the degree of consumer heterogeneity (*σ*^2^) and the degree of diseconomies of scope (*γ*).

Here, ${\bar{\sigma }}^{2}=4{\left(\sqrt{A}+\sqrt{B}\right)}^{2}-a^{2}/4$.

In case 1, when categories are substitutable, the optimal number of categories is less than when the categories are substitutable or when the categories are independent under conditions in which the product values are high or the product values are low and the degree of heterogeneity is sufficiently high, the degree of heterogeneity and the degree of scope diseconomies are both sufficiently low. However, in case 2, the optimal number of categories is greater when the categories are complements than when the categories are independent. The proposition reveals that the optimal number of categories is affected by the relationship of categories. The e-retailer increases the number of categories if categories are complementary; however, the e-retailer decreases the number of categories when categories are substitutable.

Furthermore, we investigate how the relationships of product categories affect the e-retailer’s expected profits. In the reselling mode, we answer the question in the following proposition.

**Proposition 9** (1) When categories are substitutable (*k*>0)

The e-retailer’s expected profits are lower when the categories are substitutable than when the categories are independent ($E\pi_{r}^{s}<E\pi_{r}$) if

$$0<\gamma <\frac{k{\left(\sqrt{a^{2}+4\sigma^{2}}-4\sqrt{A}\right)}^{2}}{4((\sqrt{A}-\sqrt{B})\sqrt{a^{2}+4\sigma^{2}}-2kf)}.$$

(2) When categories are complements (*k*<0)

The e-retailer’s expected profits are higher when the categories are complements than when the categories are independent ($E\pi_{r}^{s}>E\pi_{r}$) if

$$0<\gamma <\frac{-k{\left(\sqrt{a^{2}+4\sigma^{2}}-4\sqrt{A}\right)}^{2}}{4((\sqrt{B}-\sqrt{A})\sqrt{a^{2}+4\sigma^{2}}+2kf)}.$$

Proposition 9 implies the following. When the degree of diseconomies of scope that strictly depends on consumer heterogeneity is weak, the e-retailer’s expected profits are lower when categories are substitutable than when the categories are independent. However, the results are the opposite if the categories are complementary. Since the retail price is lower when products are substitutable, but the wholesale price is no lower. Moreover, when the degree of diseconomies of scope is weak, the number of categories is less when categories are substitutable than when categories are independent. Therefore, the e-retailer’s expected profits decrease. However, when the categories are complementary, it is beneficial to increase the number of categories and improve the sales volume, and the retail price is enhanced. The e-retailer’s expected profits are better when the categories are complementary than when the categories are independent. That is, the retailer’s expected profits are increasing when categories are complementary and the degree of diseconomies of scope is weak.

## 6. Indirect network effects

In the previous subsection, we investigated the impact of the direct network effects on the e-retailer’s expected profits. This section, we examine the impact of the indirect network effects on the e-retailer’s expected profits. Let *x* be the degree of indirect network effects and the other conditions remain the same. Therefore, in the reselling mode, the e-retailer’s profits and the manufacturer’s profits are respectively

$$\pi_{r}^{sI}=n(a+\varepsilon +\beta q_{i}+xn-q-k(n-1)q-w_{i})q-nf-\gamma {\left(nq\right)}^{2}/2$$ (35)

$$\pi_{mi}^{sI}=w_{i}Eq$$ (36)

Similarly, we can get the wholesale price and quantity as follows

$$w_{i}=\frac{a+nx}{2},q=\frac{a+nx+2\varepsilon }{2[2k(n-1)+2(1-\beta )+nr]}$$ (37)

Therefore, the e-retailer’s expected profits expression is

$$E\pi_{r}^{sI}=\frac{n(a^{2}+4\sigma^{2}+2anx+n^{2}x^{2})}{8[(2k(n-1)+2(1-\beta )+n\gamma ]}-nf$$ (38)

We can easy get the optimal the optimal number of categories as follows

$$\begin{array}{l} n^{***}=\frac{\left(8fH^{2}-2x(aH+3Bx)+2^{2/3}{\left(C+3\sqrt{3D}\right)}^{1/3}\right)}{6Hx^{2}}+\\ \;\frac{22^{1/3}(x(aH-3Bx)-4fH^{2}{)}^{2}}{6Hx^{2}{\left(C+3\sqrt{3D}\right)}^{1/3}} \end{array}$$ (39)

Notes

$$\begin{array}{l} C=128f^{3}H^{6}-96f^{2}H^{4}x(aH-3Bx)+24fH^{2}x^{2}{\left(aH-3Bx\right)}^{2}+\\ \;x^{3}(-2a^{3}H^{3}-9a^{2}BH^{2}x+54aB^{2}Hx^{2}-54Bx(2\sigma^{2}H^{2}+B^{2}x^{2}))\\ D=B(H^{2}x^{4}[-256f^{3}H^{4}+192f^{2}H^{2}x(aH-3Bx)-48fx^{2}{\left(aH-3Bx\right)}^{2}+\\ \;x^{3}(4a^{3}H-9a^{2}Bx+108\sigma^{2}Bx)](a^{2}H^{2}-4aBHx+4(\sigma^{2}H^{2}+B^{2}x^{2}))\\ H=2k+\gamma \end{array}$$

**Fig 1:**
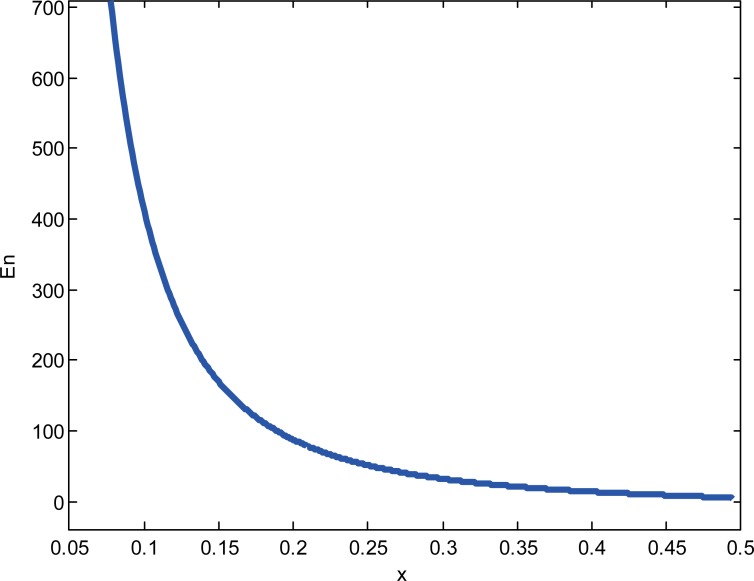
The relationship between the indirect network effects and the number of categories.

Similar to the previous subsection, we can get the optimal number of categories n*** to maximize the e-retailer’s expected profits. We investigate the impact of indirect network effects on the product variety with numerical studies. According to the assumptions, we set *a* = 6, *f* = 1.5, *σ*^2^ = 4, *γ* = 0.58, *k* = 0.2,*β* = 0.3. The result is shown in “Fig 1”. The optimal number of categories is decreasing with the indirect network externalities. In some regions, when the indirect network externalities are strong, the attractiveness is strong. Although the number of categories decreases, the e-retailer can get better expected profits. That is, when the indirect network externalities are high, it is beneficial for the e-retailer to reduce the number of categories in the reselling mode.

## 7. Numerical studies

This section provides illustrative examples for each model and reports the results of a simulation study to assess the effect of the reselling mode and the platform mode on the e-retailer’s expected profits.

### 7.1 Direct network effects

**Fig 2:**
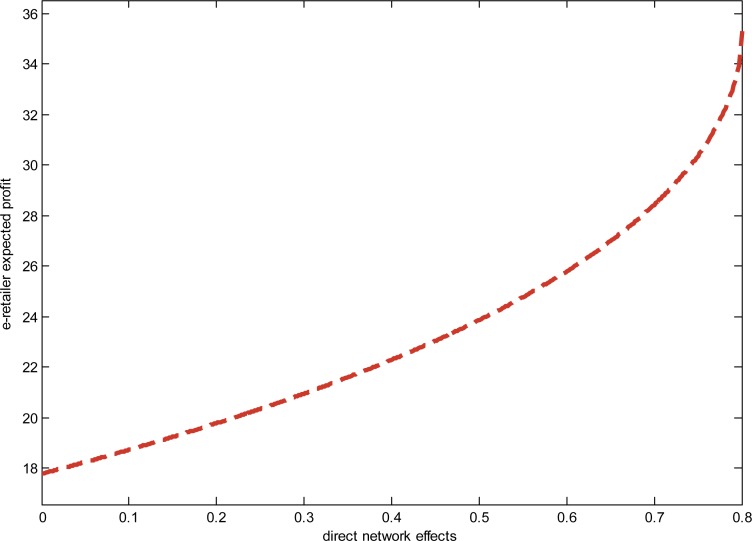
The relationship between the direct network effects and the profit.

We consider the following variants of the basic model: *a* = 15, *σ*^2^ = 1, *γ* = 0.4, *k* = 0.2, and observe the relationship between direct network effects and the e-retailer’s expected profits in reselling modes. The result is shown in “Fig 2”.

As shown in Fig 2, the e-retailer’s expected profits increase in the direct network effects. The e-retailer is better off when the degree of the direct network effects is strong.

### 7. 2 Scope diseconomies and consumer heterogeneity

**Fig 3:**
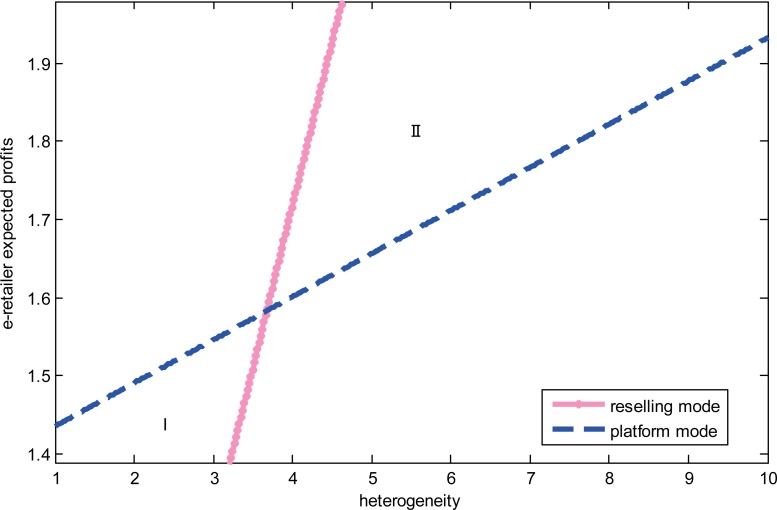
The relationship between the heterogeneity and the e-retailer’s strategies.

**Fig 4:**
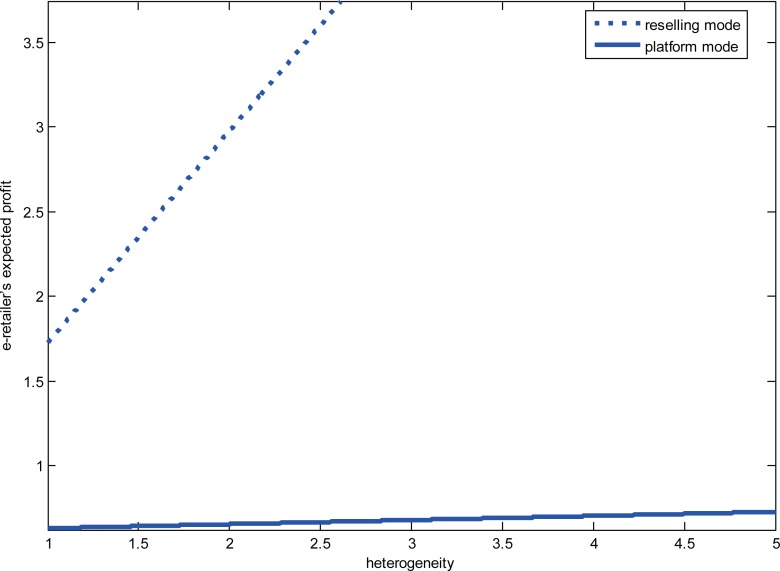
The relationship between the diseconomies of scope and the e-retailer’s strategies.

**Fig 5:**
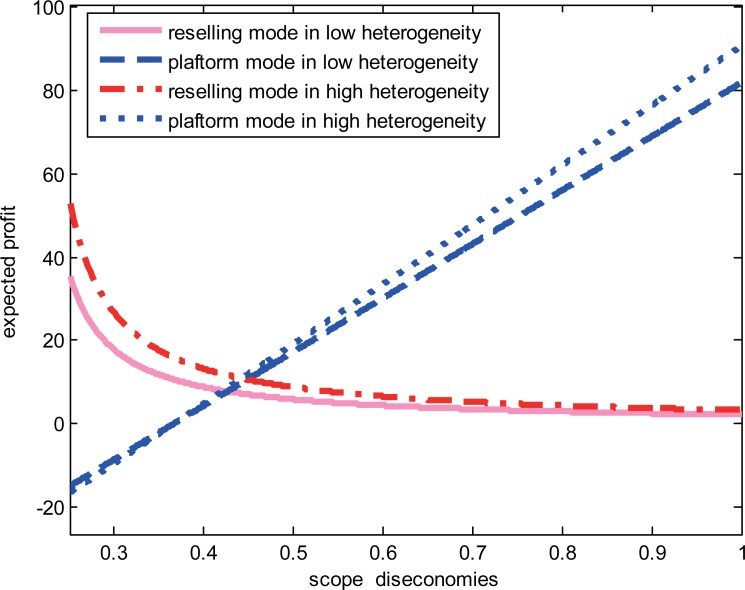
The relationship between the product’s value and the e-retailer’s strategies.

We study the impact of the diseconomies of scope and consumer heterogeneity on the e-retailer’s choice when the product categories are complements. We set *a* = 5,*f* = 1.5, *γ* = 0.7,*β* = 0.3,*k* = −0.2 and focus on the impact of consumer heterogeneity. Given high degree of diseconomies of scope when product values are low, we investigate the impact of the heterogeneity on the e-retailer’s strategies. The results are shown in “Fig 3”. When the degree of heterogeneity is higher than a set threshold, the e-retailer prefers the reselling mode; otherwise, it prefers the platform mode. When the degree of diseconomies of scope is low, we set *a* = 5,*f* = 1.5, *γ* = 0.3,*β* = 0.3 and *k* = −0.1, and the results are shown in “Fig 4”. The e-retailer’s expected profits are always higher in the reselling mode than that in the platform mode regardless of the degree of consumer heterogeneity. When the product’s value is high without the degree of consumer heterogeneity, we set *a* = 8,*f* = 1.5,*σ*^2^ = 8,*β* = 0.3,*k* = −0.1, and the results are shown in “Fig 5”. From the picture, we can find that regardless of the low-degree and the high-degree of consumer heterogeneity, the e-retailer prefers the reselling mode when the degree of diseconomies of scope is lower than a set threshold; otherwise, the e-retailer prefers the platform mode.

### 7.3 The relationship of categories

**Fig 6:**
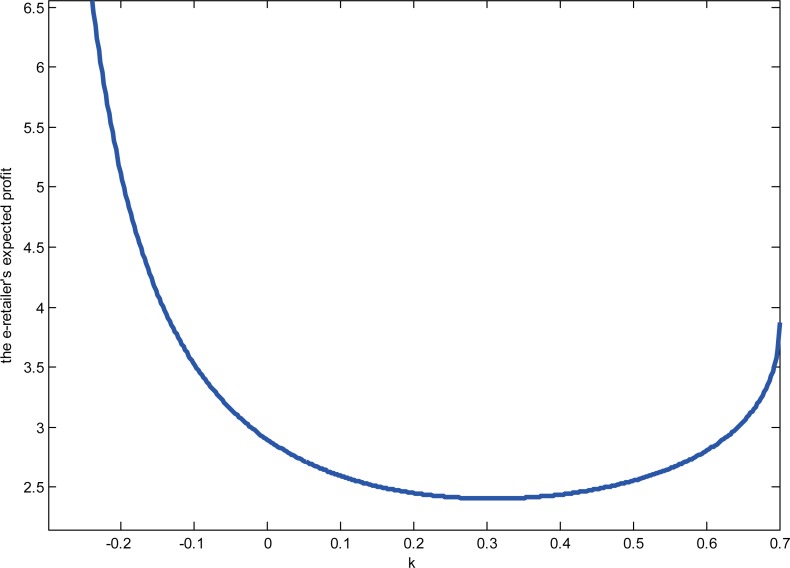
The e-retailer expected profits with *k*.

To investigate the impact of product categories’ relationships on the e-retailer’s expected profits, given the degree of diseconomies of scope, we set *a* = 8,*f* = 1.5,*σ*^2^ = 1,*β* = 0.3,*γ* = 0.5. The results are shown in “Fig 6”. The e-retailer’s expected profits decrease first and then increase in *k*.

## 8. Concluding remarks

As online platforms continue growing, major e-retailers, such as JD and Amazon, are dependent on the platform selling, and more categories will be available on the platforms. However, how to govern categories is crucial for platform owners. This paper develops several fundamental models based on consumer heterogeneity and diseconomies of scope to explore how to manage the product variety. To be specific, which product categories are adopted the reselling mode, and which product categories are adopted the platform mode. Our results have clear management insights. In brief, the e-retailer should prefer the platform mode for the following types of categories: (1) when the relationship of categories is substitutable, the product value is high or the degree of consumer heterogeneity is high, or (2) when the relationship of categories is complementary, the product value is high and the degree of diseconomies of scope is strong or the degree of consumer heterogeneity and the degree of diseconomies of scope are both high. Furthermore, the e-retailer may increase the number of categories when categories are complementary, but decrease it when categories are substitutable. In some cases, the e-retailer benefits from the consumer heterogeneity in the platform mode. These results were not found in literature [9] and [16]. This is because, we consider the diseconomies of scope and consumer heterogeneity in the model and extend their conclusions. Moreover, we take the relationship of among categories into the model. However, the literature [8] does not incorporate these in the model.

Our finding has several implications for management categories on platform. First, the e-retailer prefers platform access fee with profits sharing when consumers are heterogeneous. Second, the e-retailer prefers the platform mode to sell high value products, both enhancing the platform performance and manufacturers profits. Third, the degree of diseconomies of scope is higher, the e-retailer prefer the platform mode. Fourth, the optimal number of categories is dependent the relationship between among categories.

Our study contributes to the literature as follows: Firstly, we extend the existing literature into behavior operation and consider how consumer’s behavior affects the e-retailer’s strategies; Secondly, we show that the diseconomies of scope affects the e-retailer’s strategies and performance; Thirdly, we find that the relationship of among categories affect the platform performance and the optimal number of categories is limited.

Significant work remains open. The current model considers only a monopolistic platform. However, in practice, platforms are competitive. Therefore, it is worthwhile to extend the model here to accommodate more complicated cases, such as competing platforms and multi-homes. Another meaningful direction for future research is to introduce platform services. It may be that different platforms may offer different services, such as customer services, returns management, after-sale services and delivery options.

Funding Information:

The research was supported by the National Natural Science Foundation of China under Grants 71372140.

## Appendix: Proofs of propositions

Noted *A* = (1−*β*)*f*, *B* = (1−*β*−*k*)*f*

Proof of proposition 2

$$\begin{array}{l} \frac{E\pi_{r}}{\partial \sigma^{2}}=\frac{1}{8\gamma }>0,\frac{E\pi_{r}^{PT}}{\partial \sigma^{2}}=\frac{1}{4\gamma \sqrt{(a^{2}+4\sigma^{2})(1-\beta )f}}>0\\ \frac{E\pi_{r}}{\partial \gamma }=-\frac{1}{8\gamma^{2}}<0,\frac{E\pi_{r}^{PT}}{\partial \gamma }=-\frac{1}{8\gamma^{2}\sqrt{(1-\beta )f}}<0\\ \frac{E\pi_{r}}{\partial \beta }=\frac{f(\sqrt{\left(a^{2}+4\sigma^{2}\right)}-4\sqrt{(1-\beta )f})}{2\gamma \sqrt{(1-\beta )f}}>0 \end{array}$$

The proposition is proved

Proof of proposition 3

Proof: $E\pi_{r}^{PT}-E\pi_{r}^{PF}=(\sqrt{a^{2}+4\sigma^{2}}-4\sqrt{(1-\beta )f})[\frac{\sqrt{a^{2}+4\sigma^{2}}[\sigma^{2}+2a\sqrt{(1-\beta )f}(1-\frac{a}{\sqrt{a^{2}+4\sigma^{2}}})]}{8f\gamma (1-\beta )\sqrt{a^{2}+4\sigma^{2}}}$ Moreover, *σ*^2^>4*A*−*a*^2^/4 or *a*>4*A*.

So, we can get $E\pi_{r}^{PT}-E\pi_{r}^{PF}>0$. Hence, the e-retailer chooses the profits sharing.

The proposition is proved

Proof of proposition 4

$$E\pi_{r}-E\pi_{r}^{PT}=\frac{\begin{array}{l} \sigma^{2}{\left(\sqrt{a^{2}+4\sigma^{2}}-4\sqrt{f(1-\beta )}\right)}^{2}+\\ a^{2}(\sqrt{a^{2}+4\sigma^{2}}-3\sqrt{f(1-\beta )})(\sqrt{a^{2}+4\sigma^{2}}-4\sqrt{f(1-\beta )}) \end{array}}{8fr(-1+\beta )\sqrt{a^{2}+4\sigma^{2}}}$$

According to assumption, there is *σ*^2^>4*A*−*a*^2^/4 and *a*<4*A* or *a*>4*A*, so $\sqrt{a^{2}+4\sigma^{2}}>4\sqrt{A}$, therefore, $E\pi_{r}<E\pi_{r}^{PT}$ The proposition is proved

Proof of proposition 5

$$\frac{\partial n^{*}}{\partial \gamma }=-\frac{\sqrt{(a^{2}+4\sigma^{2})(1-\beta )f}-4(1-\beta )f}{2f\gamma^{2}}<0,\frac{\partial n^{*}}{\partial \sigma^{2}}=\frac{1-\beta }{\gamma \sqrt{(a^{2}+4\sigma^{2})(1-\beta )f}}>0$$

$$\frac{\partial n^{*}}{\partial \beta }=\frac{8\sqrt{(1-\beta )f}-\sqrt{a^{2}+4\sigma^{2}}}{4\gamma \sqrt{(1-\beta )f}},\sigma^{2}<16(1-\beta )f-\frac{a^{2}}{4},\frac{\partial n^{*}}{\partial \beta }>0,\sigma^{2}>16(1-\beta )f-\frac{a^{2}}{4},\frac{\partial n^{*}}{\partial \beta }<0$$

The proposition is proved

Proof of proposition 6

Proof: the first order of the e-retailer’s expected profits for *k* is

$$\begin{array}{l} \frac{\partial E\pi_{r}^{s}}{\partial k}=-\frac{\left(\sqrt{a^{2}+4\sigma^{2}}-4\sqrt{f(1-\beta -k)})(\sqrt{\left(a^{2}+4\sigma^{2})f(1-\beta -k\right)}+f(4b-2(2+\gamma ))\right)}{4\sqrt{f(1-\beta -k)}{\left(2k+\gamma \right)}^{2}}\\ when\;\gamma <\bar{\gamma }=\frac{\sqrt{\left(a^{2}+4\sigma^{2})f(1-\beta -k\right)}-4f(1-b)}{2f},then\;\sqrt{\left(a^{2}+4\sigma^{2})f(1-\beta -k\right)}+f(4b-2(2+\gamma ))>0 \end{array}$$

So, $\frac{\partial E\pi_{r}^{s}}{\partial k}<0$

$$\frac{\partial E\pi_{mi}^{s}}{\partial k}=\frac{a^{2}(a^{2}+4\sigma^{2})f^{2}}{4{\left[(a^{2}+4\sigma^{2})f(1-\beta -k)\right]}^{3/2}}>0$$

The proposition is proved

Proof of proposition 7

$$E\pi_{r}^{s}-E\pi_{r}^{ps}==\frac{-((\sqrt{a^{2}+4\sigma^{2}}-4\sqrt{B})(2\gamma \sigma^{2}(\sqrt{a^{2}+4\sigma^{2}}-4\sqrt{B})+a^{2}(H\sqrt{a^{2}+4\sigma^{2}}-6\gamma \sqrt{B})))}{8H((a^{2}+4\sigma^{2})k+2\gamma \sqrt{(a^{2}+4\sigma^{2})B})}$$

The *σ*^2^>4*f*(1−*β*−*k*)−*a*^2^/4, then $E\pi_{r}^{s}<E\pi_{r}^{ps}$

(1) When *k*>0,

If $0<\gamma <\frac{-k\sqrt{\left(a^{2}+4\sigma^{2}\right)}}{\sqrt{f(1-\beta -k)}}$, then $E\pi_{r}^{s}<E\pi_{r}^{ps}$; If $\gamma >\frac{-k\sqrt{\left(a^{2}+4\sigma^{2}\right)}}{\sqrt{f(1-\beta -k)}}$, then $E\pi_{r}^{s}<E\pi_{r}^{ps}$.

The proposition is proved.

Proof of proposition 8

$$\begin{array}{l} n^{**}-n^{*}=-\frac{\sqrt{a^{2}+4\sigma^{2}(}(2k+\gamma )\sqrt{(1-\beta )f}-\gamma \sqrt{f(1-\beta -k)})-4fk(2-2\beta +\gamma )}{2f\gamma (2k+\gamma )}\\ =-\frac{(2k\sqrt{A}(\sqrt{a^{2}+4\sigma^{2}}-4\sqrt{A}))+(\sqrt{a^{2}+4\sigma^{2}}(\sqrt{A}-\sqrt{B})-4fk)\gamma }{2f\gamma (2k+\gamma )}\\ when\;k>0,If4\sqrt{A}<a<4(\sqrt{A}+\sqrt{B}),\sigma^{2}>{\bar{\sigma }}^{2}=4{\left(\sqrt{A}+\sqrt{B}\right)}^{2}-a^{2}/2,n^{**}<n^{*}\\ 0<\sigma^{2}<{\bar{\sigma }}^{2},0<\gamma <\frac{2k\sqrt{A}(\sqrt{a^{2}+4\sigma^{2}}-4\sqrt{A})}{4fk-\sqrt{a^{2}+\sigma^{2}}(\sqrt{A}-\sqrt{B})},n^{**}<n^{*}\\ If\;a>4(\sqrt{A}+\sqrt{B}),n^{**}<n^{*}\\ when\;k<0,If4\sqrt{B}<a<4(\sqrt{A}+\sqrt{B}),\sigma^{2}>{\bar{\sigma }}^{2}=4{\left(\sqrt{A}+\sqrt{B}\right)}^{2}-a^{2}/2,n^{**}>n^{*}\\ 0<\sigma^{2}<{\bar{\sigma }}^{2},0<\gamma <\frac{-2k(\sqrt{A((a^{2}+4\sigma^{2})}-4A)}{\sqrt{a^{2}+4\sigma^{2}}(\sqrt{A}-\sqrt{B})-4fk},n^{**}>n^{*}\\ If\;a>4(\sqrt{A}+\sqrt{B}),n^{**}>n^{*} \end{array}$$

The proposition is proved

Proof of proposition 9

$$\begin{array}{l} E\pi_{r}^{s}-E\pi_{r}=-\frac{1}{4r(2k+r)}[k(\sqrt{\left(a^{2}+4\sigma^{2}\right)}-4\sqrt{A}{)}^{2}+4\gamma (\sqrt{\left(a^{2}+4\sigma^{2}\right)}(\sqrt{B}-\sqrt{A})+2kf)]\\ when\;k>0,\sqrt{\left(a^{2}+4\sigma^{2}\right)}(\sqrt{B}-\sqrt{A})+2kf<0\\ 0<\gamma <\frac{k{\left(\sqrt{a^{2}+4\sigma^{2}}-4\sqrt{A)}\right)}^{2}}{4((\sqrt{A}-\sqrt{B})\sqrt{a^{2}+4\sigma^{2}}-2kf)},E\pi_{r}^{s}<E\pi_{r};otherwise\;E\pi_{r}^{s}>E\pi_{r}\\ when\;k<0,\sqrt{\left(a^{2}+4\sigma^{2}\right)}(\sqrt{B}-\sqrt{A})+2kf>0\\ 0<\gamma <\frac{-k{\left(\sqrt{a^{2}+4\sigma^{2}}-4\sqrt{A}\right)}^{2}}{4((\sqrt{B}-\sqrt{A})\sqrt{a^{2}+4\sigma^{2}}+2kf)},E\pi_{r}^{s}>E\pi_{r} \end{array}$$

The proposition is proved
